# ChatGPT and Other Large Language Models in Medical Education — Scoping Literature Review

**DOI:** 10.1007/s40670-024-02206-6

**Published:** 2024-11-13

**Authors:** Alexandra Aster, Matthias Carl Laupichler, Tamina Rockwell-Kollmann, Gilda Masala, Ebru Bala, Tobias Raupach

**Affiliations:** Institute of Medical Education, University Hospital Bonn, Venusberg-Campus 1, 53127 Bonn, Germany

**Keywords:** ChatGPT, Large language model, Artificial intelligence, Generative AI, Medical education

## Abstract

This review aims to provide a summary of all scientific publications on the use of large language models (LLMs) in medical education over the first year of their availability. A scoping literature review was conducted in accordance with the PRISMA recommendations for scoping reviews. Five scientific literature databases were searched using predefined search terms. The search yielded 1509 initial results, of which 145 studies were ultimately included. Most studies assessed LLMs’ capabilities in passing medical exams. Some studies discussed advantages, disadvantages, and potential use cases of LLMs. Very few studies conducted empirical research. Many published studies lack methodological rigor. We therefore propose a research agenda to improve the quality of studies on LLM.

## Introduction

### The Rise of Large Language Models

For several years now [1, 2], high-performance artificial intelligence applications capable of understanding and generating natural language [3] have been existing, which are collectively referred to as “large language models” (LLMs). However, the general population became aware of this development mostly after November 30, 2022, when the freely accessible LLM “Chat Generative Pre-trained Transformer” (abbreviated as ChatGPT) was released by OpenAI [4]. ChatGPT amazes many users because it generates responses that are often indistinguishable from those of humans. While many other LLMs from various providers were released shortly after the launch of ChatGPT [5], particularly in popular media, ChatGPT is most popular in many parts of the world.

ChatGPT and other LLMs have already had a significant impact on various research fields in the first months since their release. Examples can be found in the most diverse fields, including software engineering [6], journalism [7], and finance [8]. Two areas stand out particularly, with practitioners in both domains expecting a great benefit from the use of LLMs: healthcare [9] and education [10]. In the healthcare sector, researchers currently anticipate promising application opportunities across three key areas [9, 11]: medical research (e.g., scientific text production and science communication), patient care/clinical practice (e.g., patient empowerment and improved documentation), and medical education. Educators, on the other hand, anticipate a positive impact of LLMs on learning processes (e.g., providing real-time feedback and improving students’ writing styles) as well as on teaching processes (e.g., planning lessons and enhancing assessment) [10]. Besides the manifold possibilities of LLMs, representatives from both disciplines also point out potential risks associated with their use. For example, Omiye et al. [12] found that many LLMs propagate “harmful, inaccurate, race-based” medicine [12]. In the field of education, other potential practical and ethical limitations have been discussed, such as the lack of replicability of results [13].

Apparently, the use of LLMs is being discussed intensely in both the medical and education domains, raising questions about the influence of LLMs on the intersection of the two fields: medical education.

### LLMs in Medical Education

An example that has garnered significant attention in the medical community in recent months, fueling considerations regarding the use of LLMs in medical education, was ChatGPT’s ability to pass high-stakes medical examinations. Kung et al. [14] demonstrated relatively early on that ChatGPT can pass the United States Medical Licensing Examination (USMLE). Results like these have led other researchers to assert that these findings shine “a spotlight on the flaws of medical education” [15] as it may not require a human brain and mind to pass these exams. However, beyond the remarkable exam performance of ChatGPT, medical educators also seem to have recognized that AI-assisted LLMs like ChatGPT can have a significant positive as well as negative impact on the training of future physicians [16]. Yet, attempting to gain an overview of published research findings on this topic leads to a research landscape characterized by uninformed statements and questionable methods.

### Aims and Scope

While a multitude of review papers on the use of AI in medical education already exist at the time of writing [17, 18], this review aims to provide a brief summary of the insights gained from 1 year of using LLMs such as ChatGPT in medical education. Given that the availability of ChatGPT and other chatbot LLMs offers a low-threshold entry point for the use of AI in medical education, we anticipate that AI-supported educational applications will play an increasingly significant role in medical schools.

Thus, this review aims to provide a comprehensive overview of the state of the literature on the use of LLMs in medical education, precisely 1 year after the publication of ChatGPT. Our main objective was to investigate the level of scientific interest (measured by the number of scientific publications) in LLMs in medical education shortly after the publication of the first freely accessible LLMs. We also wanted to examine the rough thematic foci and publication outlets (e.g., journals, conferences) of the articles as well as the global distribution of the associated research papers. Therefore, our first research question was:

RQ1: What is the extent of publications regarding LLMs in medical education 1 year after the release of the most prominent LLM, ChatGPT?

The next logical step is the examination of themes addressed in publications on LLMs in medical education. As mentioned earlier, some researchers appear to focus on ChatGPT’s capabilities in answering medical exams. Others, however, utilize LLMs to generate new exam questions [19]. Additionally, there is a plethora of opinion pieces balancing the benefits of implementing LLMs in medical schools against potential limitations [20]. An overview of the addressed themes would be helpful for medical educators, as it would enable them to easily identify what has already been done and which areas for future research appear most promising. Therefore, research question 2 was as follows:

RQ2: What specific themes do publications exploring the utilization of LLMs in medical education focus on?

Given the short time frame between the advent of LLMs (i.e., November 2022) and publication dates (up until November 2023), one could critically question the empirical quality of articles that plan, conduct, evaluate, and publish a complete study within but a few months. It is therefore reasonable to assume that a large portion of the publications is not based on empirical findings but rather on conceptual and theoretical considerations. While conceptual articles certainly have their value, it is still important to examine the ratio of the different types of publications. Hence, research question 3 read as follows:

RQ3: What is the relation of empirical research to theoretical/conceptual publications regarding LLMs in medical education?

Finally, it would also be helpful to know which medical specialties are taking on pioneering roles in the field of LLM in medical education research. Since LLMs belong to the family of natural language processing AI applications, it could be assumed that conversation-heavy disciplines such as psychotherapy, psychiatry, and to some extent even family medicine have a particularly keen interest in the use of LLMs in their teaching.

RQ4: Which medical specialties are currently involved in the exploration of LLMs within medical education?

## Method

In conducting this scoping literature review, we adhered to the recommendations outlined in the “Preferred Reporting Items for Systematic Reviews and Meta-Analyses extension for Scoping Reviews (PRISMA-ScR)” by Tricco et al. [21].

### Eligibility Criteria

We applied a total of six eligibility criteria for selecting appropriate publications (see Table 1). These criteria can be understood as hierarchically organized rules, whereby we first assessed coverage of the first eligibility criterion for each report. If this criterion was met, the second criterion was examined, and so forth. Only reports meeting all six eligibility criteria were included in the review.

**Table 1:** Eligibility criteria

No	Eligibility criteria	Exclusion criteria
1	Published after Jan 1, 2000^1^	Published before Jan 1, 2000^1^
2	Abstract and main text were published in English or German	Abstract and main text were published in another language
3	Published in a scientific journal or as a conference paper at a scientific conference or as part of a scientific book/collection	Published in other outlets such as newspapers, magazines, websites, social media, and non-scientific books
4	Focused on the application or influence of ChatGPT or another LLM (Bard, Bing Chat, etc.)	Report is not focused on LLMs
5	Focused solely on academic and vocational healthcare profession education (medical education, nursing education, continuing medical education, etc.)	Focused on education in other disciplines (engineering education, legal education, etc.)
6	Focused on education (in healthcare professions)	Not focused on education (e.g., using ChatGPT for clinical practice or medical research)

The same eligibility criteria were employed in both steps of the review process (i.e., abstract and full-text assessment, see section “Search Strategy”). Eligibility criterion 3 refers solely to the type of scientific outlet the report was published in, not on the article type (research articles, opinion pieces, etc.)

^1^While AI-supported natural language processing, and particularly large language models (LLMs), has only gained relevance in the past few years, we chose the year 2000 as the starting point to ensure that we did not overlook any potential pioneering articles. Additionally, there were instances where the databases (e.g., PubMed) listed incorrect publication dates.

### Search Strategy

**Fig. 1:**
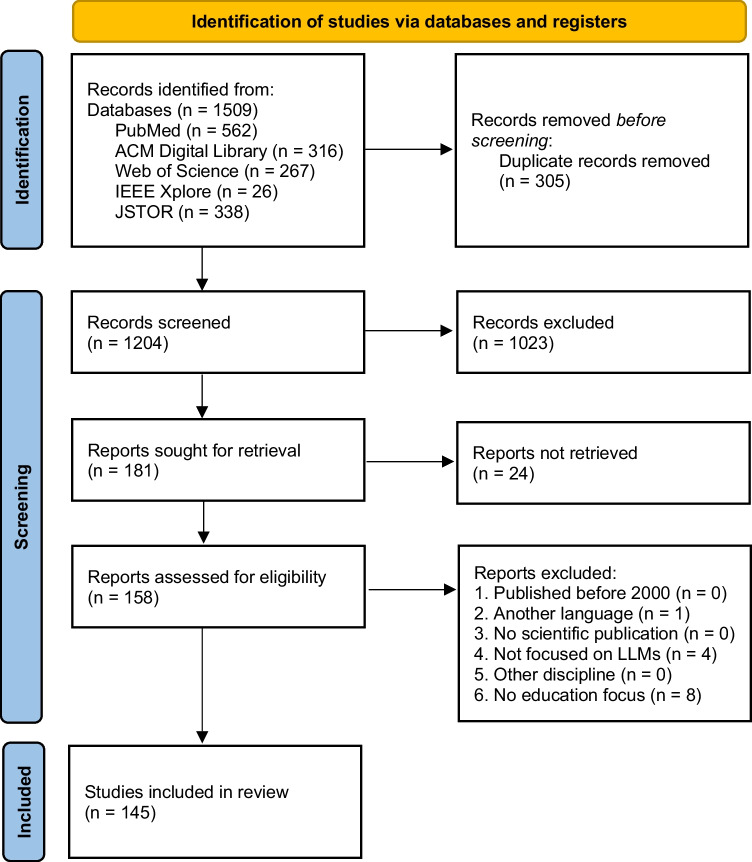
Flow diagram of the literature review process. The figure, which was adapted from Page et al. [22], was slightly modified, since we relied exclusively on databases and registers for study identification

We searched the following scientific literature databases: ACM Digital Library (Association for Computing Machinery), IEEE Xplore (IEEE), JSTOR (ITHAKA), PubMed (National Library of Medicine), and Web of Science (Clarivate). We did not search other databases and did not use search engines to find gray literature. However, due to the novelty of the topic, we did include preprints that have not yet been peer-reviewed, as long as they were listed in one of the databases mentioned above. We conducted the search on November 30, 2023, exactly 1 year after the publication of OpenAI’s ChatGPT platform. Fig. 1 provides a more detailed breakdown of the number of records retrieved from the respective databases. We used the following combination of search terms and boolean operators in each of the five databases: (“LLM” OR “LLMs” OR “large language model*” OR “GPT” OR “ChatGPT” OR “chatbot*”) AND (“medical education” OR “medical school*” OR “medical exam*” OR “medical assessment*” OR “medical curricul*” OR “healthcare education” OR “continuing medical education” OR “internship” OR “residen*”).

### Review Strategy

We employed a two-step literature review strategy. In step 1, we used Rayyan (rayyan.ai) [23], a software designed to help with organizing and conducting literature reviews. We retrieved the search results from all databases and registers, encompassing titles, abstracts, and bibliographic details, and subsequently imported the generated files into Rayyan. Following this, two authors (AA and GM) conducted an independent evaluation of each report based on the predefined exclusion criteria (see section “Eligibility Criteria”). During the evaluation stage, each rater was blinded to the decision of the other rater. Once all abstracts had been assessed, the blind-review mode was lifted, and any disparities were addressed and resolved through systematic discussion. In step 2, we sought to retrieve all reports whose titles and abstracts indicated that they were eligible for inclusion. Two authors (AA and TRK) independently read the remaining reports in their entirety and checked them against the exclusion criteria. The decisions were subsequently compared, and discrepancies were discussed under the moderation of another author (MCL). Inter-rater reliability, expressed as Cohen’s kappa, was ϰ = 0.911, which can be interpreted as nearly perfect agreement [24]. We calculated Cohen’s ϰ using the online calculator provided by Hemmerich [25]. Finally, a content analysis table was developed to extract the most relevant information from the studies, which were needed to answer the four research questions. The content analysis table consisted of several questions (organized in columns), each referring to a specific characteristic of the respective paper (organized in rows). For instance, the table included questions about the journal in which the paper was published or the thematic focus on which the paper concentrated. Due to the large number of included studies, the completion of the table was divided between authors, with the first and second halves of the entries being completed by two authors (AA and TRK for the first half and MCL and EB for the second half, respectively). Thus, the content of each study was independently analyzed by two authors. The content analysis procedure was conducted in Microsoft Excel (Microsoft, Version 2016), and data visualization was done via RStudio (Posit Software, Version 2023).

## Results

### Selection of Sources of Evidence and RQ 1: Number of Publications

Across all databases, a total of 1509 records were obtained of which 305 (20%) were excluded due to being duplicates. The titles and abstracts of 1204 (80%) records were subsequently checked for eligibility. After the exclusion of 1021 (68%) records, the full texts of 181 (12%) records were sought for retrieval. Since 24 (2%) records were not retrievable, the remaining 158 (10%) reports were assessed for eligibility. The most frequent reason for exclusion was reason 6 (see Table 1) with eight reports that had to be excluded, followed by reason 4 (*n* = 4) and reason 2 (*n* = 1). Consequently, 145 articles (10%) were included in the review (see Fig. 1) [14, 15, 20, 26–166].

The distribution of the publications encompassed a total of 80 journals and two preprint servers. The largest number of studies was published in *JMIR Medical Education* (*n* = 19), directly followed by *Cureus* (*n* = 18). Few studies were published in leading medical education journals such as *Academic Medicine* (*n* = 5), *Medical Education* (*n* = 1), or *Medical Teacher* (*n* = 1). In the majority of journals, only one publication was found. Seven reports were found on preprint servers (arXiv.org and medrXiv.org).

**Fig. 2:**
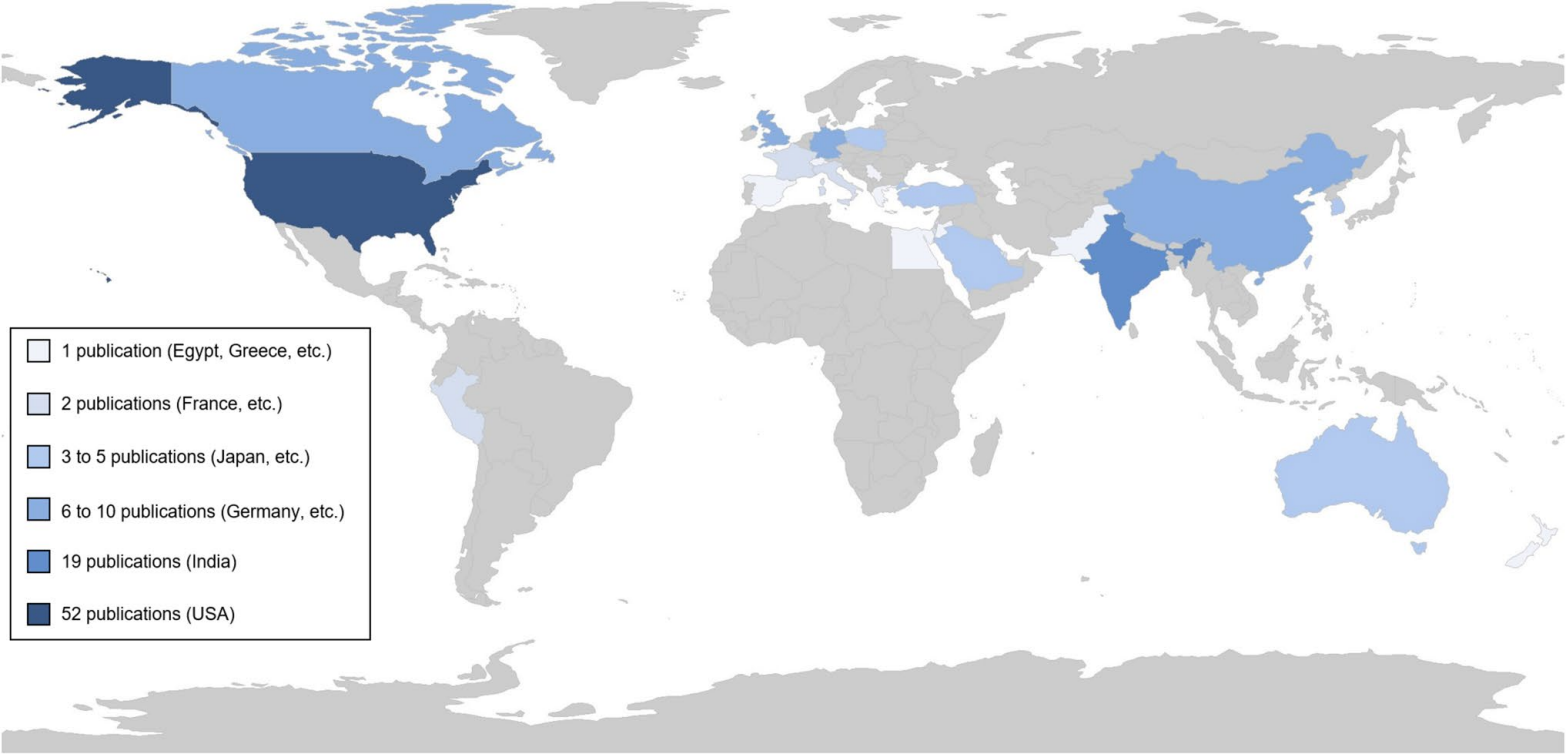
Map of the corresponding authors’ origin. We found no publications from authors of countries highlighted in gray. A deeper shade of blue indicates more publications on LLMs in medical education; a lighter shade of blue indicates less (but not zero) publications

The corresponding authors of the publications came from 29 different countries (see Fig. 2). The countries with the most publications were the USA (*n* = 52, 36%) and India (*n* = 19, 13%).

### RQ 2: Thematic Foci

In order to gain an overview of the thematic foci of the studies, we assigned each study to an overarching focus (see Table 2 presenting an overview of the foci including the references). The majority of studies (*n* = 75, 52%) focused on demonstrating ChatGPT’s capabilities in passing medical exams (e.g., USMLE, NMLE of different countries) or answering board exam preparation questions (e.g. neurosurgery oral board preparation question bank [70]). Other studies assessed ChatGPT’s performance in medical school-specific exams (e.g., dermatology [59], orthopedics [152], ophthalmology [87]). Across all exams, ChatGPT-4 seems to perform better than ChatGPT-3.5. For more information on the general distribution across medical specialties, please refer to Table 3.

**Table 2:** Overview of the thematic foci

Foci		Reference
Empirical	Non-empirical	
Empirical research based on collected data (qualitative or quantitative) without research on ChatGPT’s capabilities in passing medical exams (*n* = 24)		[32–34, 41, 51, 57, 76, 77, 56, 94, 96, 110, 115, 117, 119, 142, 145, 146, 151, 153, 154, 156, 161, 164]
Demonstrating ChatGPT’s capabilities in passing medical exams (*n* = 75)		[14, 26, 27, 31, 49, 50, 55, 60–66, 68–74, 80, 82, 84, 86–89, 91, 95, 97–109, 111–114, 116, 120, 122–124, 127–129, 131, 133, 135–139, 141, 143, 144, 147–150, 152, 155, 157, 159, 160, 165]
	Conceptual papers developing possible scenario cases for LLMs in medical education (*n* = 37)	[15, 28, 30, 35–37, 39, 40, 42–48, 52, 53, 58, 75, 78, 79, 81, 83, 85, 90, 92, 93, 118, 121, 125, 130, 132, 134, 140, 158, 162, 163]
	Others (*n* = 9)	[20, 29, 38, 54, 56, 59, 67, 126, 166]

**Table 3:** Number of publications across various medical specialties

Medical specialty	Absolute number	Relative number
Several/not specified	67	46%
Ophthalmology, including neuro-ophthalmology	10	7%
General surgery	6	4%
Physiology	5	3%
Neurosurgery; emergency medicine (including advanced cardiac life support, medical intensive care, and resuscitation life support)	4 each	3%
Anesthesiology; biochemistry; plastic surgery; otolaryngology	3 each	2%
Anatomy; dermatology; general medicine; internal medicine and surgery; medical microbiology; neurology and neuroscience; nursing; obstetrics and gynecology; orthopedics; orthopedic surgery	2 each	1%
Bioethics; biomedicine; biostatistics; cardiology; clinical informatics; clinical toxicology; gastroenterology; hematology; molecular medicine; parasitology; pediatric genitourinary; pharmacology; public health; radiology; radiation oncology; thoracic surgery; urology	1 each	1%

Medical specialties are based on the term used by the respective authors. Different medical specialties are separated by semicolons

The second most common category under which studies could be subsumed was conceptual papers (*n* = 37, 26%) that focus on possible scenarios in which LLMs can be used in medical education. These publications discussed opportunities, advantages, disadvantages, and possibilities for the application of LLMs in different medical fields or more generally how LLMs can be used in medical education. Most authors reflect or discuss potentials simultaneously with pitfalls [46–48, 78, 79, 93, 121]. One example of a potential pitfall mentioned by the authors of the studies is the possibility of using ChatGPT to cheat [90, 92]. The advantages mentioned by the authors include, for example, the possibility of automatically creating exams or assigning grades and feedback [75, 90]. Regarding the use for medical students, ChatGPT seemed to function as a learning resource providing a personalized learning experience, or reference work as well as a helpful tool for summarizing studies [83, 132]. In the field of medical education, the authors proposed that ChatGPT may lend itself for simulating patients adequately enough to run a comprehensive anamnesis [118]. Moreover, ChatGPT could be used to assist the writing process of recommendation letters in terms of avoiding implicit biases [140], rating recommendation letters [30], or generating students’ personal statements [28]. Other authors presented conversations in the form of a dialogue conducted with ChatGPT [36, 39, 125, 130]. These conceptual papers are mostly published in the form of editorials, letters to the editor, etc. For more information on the ratio between empirical and conceptual research, refer to RQ 3.

Another category of studies (*n* = 24, 17%) focused on empirical research based on collected data in either a qualitative or quantitative manner. The aims of the empirical studies were manifold: Some authors used ChatGPT to generate or rate already generated multiple-choice questions [34, 94, 161, 164], or compared the performance of different LLMs on written surgical case scenarios [56]. Other authors compared ChatGPT’s and students’ performance in a parasitology examination [156], or used ChatGPT as a tool for students to check their own responses against ChatGPT’s responses on complex pediatric case vignettes [76]. Another study comparing characteristics of ChatGPT’s responses to surgical problems with short summaries from textbooks found that students rated evidence-based textbook summaries better in regard to comprehensiveness, but ChatGPT’s summaries better in regard to clarity and organization [33]. Another study focused on the empirical comparison of ChatGPT’s performance on state examination questions from different countries [51]. The authors found significant differences with ChatGPT performing best for the Italian and worst for the French examination [51]. Further studies used ChatGPT for writing personal statements [153] and comparing it to statements written by humans [41]. Additionally, a study assessed the general usage of ChatGPT and the attitudes of faculty member towards it in a medical school in Antigua [32] while other studies developed surveys to assess the use of LLMs in the context of medical education [145, 151].

Lastly, *n* = 9 (6%) studies focused on other topics that were not covered by the three previous categories. This category includes systematic or scoping reviews, responses to commentaries, and other articles.

### RQ 3: Ratio Between Empirical and Conceptual Research

Regarding the included empirical research, the majority of studies (*n* = 86, 59%) took a quantitative approach mostly focusing on ChatGPT’s ability to pass medical exams (*n* = 68, 79%). Besides, seven studies represented qualitative empirical research (5%) and four studies were literature reviews (3%). The second largest amount of studies (*n* = 48, 33%) were narrative articles such as conceptual or opinion pieces (letter to the editor, editorials, etc.). Therefore, the ratio between conceptual and empirical research is 1.8:1.

### RQ4: Medical Specialties

As can be seen in Table 3, a variety of medical specialties was currently engaged in using LLMs during the first year after the introduction of ChatGPT. All studies that specified the medical specialty concentrated solely only on the reported specialty while no study covered multiple specialties.

## Discussion

### General Discussion

This scoping review aimed at assessing the current status of the use of LLMs in medical education 1 year after the launch of the best known LLM, ChatGPT. To do so, we pursued the areas of four research questions: the extent of publications, thematic foci, the ratio between empirical research, and conceptual publications, as well as the spectrum of medical specialties. In line with the preceding literature regarding the use of LLMs in healthcare [9] as well as in education [11], our results emphasized that LLMs are used in medical education at larger scale. Specifically, the 145 included studies mostly focused on ChatGPT’s capabilities in passing medical exams, followed by conceptual research concentrating on advantages, disadvantages, and possible application scenarios of LLMs in medical education. Besides the assumption that healthcare and education anticipate the use of LLMs in a practical manner, our results showed that the trend of the included studies was on conceptual ideas of possible applications for LLMs. Only 24 of the included studies used an experimental study design (either qualitative or quantitative). The utilization of LLMs was restricted to a particular medical specialty but extended across various specialties from more theoretical ones (e.g., biostatistics) to more practical ones (e.g., thoracic surgery). These results showed that LLMs are not primarily used in conversation-heavy disciplines such as psychotherapy and psychiatry, but rather in data-heavy and practical disciplines.

### Limitations

Several limitations may affect the validity of the findings. Firstly, a significant portion of the included studies was categorized as quantitative empirical research, even though no empirical study in the sense of a randomized controlled trial was conducted. This is due to the fact that most studies thematically focused on ChatGPT’s capability to pass medical exams. While these studies indeed presented quantitative data, they often did not follow a traditional study design (some intervention/manipulation, comparison with a control group, etc.). While the ability of LLMs to pass medical exams is certainly impressive, the contribution of these studies to medical education research and practice is questionable.

Another point to consider is the heterogeneous landscape of studies being published rapidly across various journals. In the span of 1 year, more than 145 studies on the topic were published in 80 different journals. Only five included studies were published in reputable journals like *Academic Medicine*, while no study was published in *Medical Teacher*, *Medical Education*, or *Advances in Health Sciences Education*. Although the focus of this review was on the use of LLMs in medical education, studies were frequently published in specialty-specific journals (e.g., *American Journal of Gastroenterology*, *British Journal of Anaesthesia*) having a high impact in the respective specialty. It would therefore be reasonable to wonder whether the associated rapid peer review and publication process could have impaired the methodological rigor and quality of the research. This assumption is also supported by the fact that few studies employed a rigorous study design, but primarily captured ChatGPT’s (mostly superficial) performance in medical exams.

However, in conducting our literature review, we also had to make some decisions that, although crucial to the feasibility of the study, could potentially limit the validity of the results. First, we refrained from explicitly searching for gray literature as we set the focus to studies published in peer-reviewed journals to ensure the inclusion of reliable high-quality primary studies. Nevertheless, we included some preprints assuming that these studies (though maybe with changes) will be published in the near future. Furthermore, in our search terms, we did not include other typical commercial LLMs such as Google’s “Bard” (later “Gemini”) or Microsoft’s “Copilot,” in addition to OpenAI’s ChatGPT. This decision was based on several factors, the primary one being that the majority of studies either utilized ChatGPT directly or referred to the generic term “large language model”. Since several articles were not retrievable, this may have affected the results of this review. Nonetheless, the sheer number of studies included suggests that the status of the current literature landscape is adequately depicted. Two independent reviewers rated each article, though not consistently by the same pair due to the number of included studies. While this might have affected the consistency of ratings, the interrater reliabilities showed satisfactory agreement values, suggesting that choosing this method was a reasonable decision.

### Implications for Future Research and Practice

Conclusively, we propose some implications for future research from our findings. It became evident that most studies assessed LLM’s (particularly ChatGPT’s) performance in medical exams. We identified the research gap that studies are mostly lacking a theoretical valid research question as well as a sound empirical approach. While it is important to understand LLM’s medical proficiency level, drawing definite conclusions becomes challenging when studies lack an empirical approach and merely engage a LLM in question-answering tasks. As a consequence of the research gap identified, strengthening the empirical approach seems to be the most important implication for future research. Particular attention should be paid to developing a theory-driven research question which contains a sound-dependent variable that is assessed by suitable measurement methods. Measurement methods as well as the evaluation of the study should be aligned to Kirkpatrick’s four-level evaluation model to assure the effectiveness of the study design. A randomized controlled pretest–posttest (true experimental) [167] that compares an experimental group to a control group should be capable of providing reliable outcomes and analyzing causal relationships. Accordingly, a group of medical students learning with a LLM could be compared with a control group of medical students learning with traditional methods (e.g., textbook case vignettes).

LLMs used in educational settings, such as medical education, should meet several requirements. First and foremost, LLMs should be able to provide their users with correct information that should be as up-to-date as possible. In the field of medical education, it is essential that LLMs do not only provide correct information but also supplement it with medical specialized information. Therefore, future studies should also look at the development of medicine-specific LLMs, such as MedPaLM2. Another possibility to harness the benefits of LLMs for medical education might be using customizable LLM-interfaces, such as ChatGPT’s new “GPT”-creation feature, in which users (e.g., medical educators) can customize their own versions. In this context, it is thinkable of GPTs customized exclusively for the use in medical education.

Regarding the support of medical teachers, LLMs could be used to generate exam questions whose quality should be constantly rated by experts in the field of medical education as well as by medical students or residents (17). It is also thinkable that LLMs can be further used to provide medical students with feedback on essays or free-text responses. Thus, it must be investigated whether the feedback was correct and valuable, and whether it has an impact on students’ learning outcomes. Besides the support of medical teachers or students, LLMs could also be used to enhance practical skills, exemplarily by providing prompts that describe a step-by-step-instruction for specific procedures, such as CPR. Additionally, other generative AI applications that are able to generate images and videos instead of text may provide further opportunities for future research. It is conceivable that generative AI may be used for medical imaging specialties education, exemplarily the generation of X-ray images for pneumothorax.

Hence, future studies should address the overarching question of how LLMs can and should be employed in medical education. Should LLMs be used to relieve or support medical teachers or can students rely on LLMs to accompany or even enhance their learning process?

### Conclusion

This scoping review shed a light on the literature landscape dealing with the use of LLMs in medical education. Our results showed that although a large number of studies were published within a year since the implementation of ChatGPT, only few studies applied a rigorous study design. Thus, we call on medical education and LLM researchers to conduct more studies following a stringent and methodologically sound research design in order to answer meaningful research questions.

## Data Availability

The datasets used and/or analyzed during the current study are available from the corresponding author on reasonable request.

## Abbreviations

AI: Artificial intelligence
ChatGPT: Chat Generative Pre-trained Transformer
LLM: Large language model
